# To Dr. Batty

**Published:** 1801-12

**Authors:** A. Carlisle

**Affiliations:** Soho Square


### Medical and Phyfical Journal.

VOL. VI.] DECEMBER, 1801. [NO. XXXIV.

### To Dr. BATTY.

DEAR SIR,

I Beg the favor of a place in your Journal, to anfwer, and endeavour to rectify, fome mifapprehenfions contained in the letter of Mr. Wilkinfon upon Fractures. The pains which that gentleman has taken to gratify the public, refpecting my queries, and the handfome manner he adopts, deferve a refpectful notice. Without any defire to occupy your Journal in trifling controverfy, I truft Mr. Wilkinfon and your readers may feel fatisfied with the following explanations.

Firft, My obfervations were quite general, and the cafes related, only intended as points from whence the principal queries fhould iffue.

Secondly, The leading queftion, requiring to be anfwered, was, On what local circumftances, or ftate of conftitution, does the deficiency of hardening matter (phofphat of lime) depend, in cafes of imperfect union in fractures generally?

Mr. Wilkinfon feems to have returned thofe incidents upon me, which were related among the prominent features of the three cafes, and has preffed them forward as fufficient caufes of the phenomena; this is only carrying the argument farther than I thought neceffary.

The failor's cafe was that which he alludes to, and admits of all the conjectures applied to it; although neither fymptoms of fcurvy, nor mollities offium in the other bones, were prefent; and I believe that Mr. I. Hunter had agreed with Mr. Lynn about the probable fuccefs of his operation.

When I ftated that no decided inference can be drawn from thefe cafes, as to the certain caufes of fuch misfortunes, I did not mean to comprehend thofe in which the inferences were already fo far drawn by myfelf. I intended to folicit further information from the relation of more cafes, fo that furgeons might be able to judge, a priori, of the chances which might favour the defective union of broken bones.

The carpenter's cafe was only defcribed to convey the general effect of its character; his fore-arm refted in a fling at right angles with the upper-arm, and the bone of the fractured brachea was kept ftrait by fplints and bandages. Whilft the broken part of the arm continued ſtraight and free from pain, I judged it in a favourable condition for union; nor did I at all ſuſpect that the tone of the muſcles had ſo far ceaſed as to admit of that ſeparation by the weight of the lower arm, which would prevent the union. I related thoſe facts to caution others againſt thoſe hopes, and that ideal ſecurity which diſappointed me in this inſtance, and which I believe would have taken the ſame courſe in every other practitioner's hands. The reader will perceive, that in all the leading points Mr. Wilkinſon and I do not differ, although on the ſubject of depletion we may vary in the general acceptation.

That inflammation is one of the conſequences of fractures to be managed with nice attention, every one muſt agree. To regulate the degree and courſe of this natural proceſs, by means of medicine, diet, &c. every one muſt alſo aſſent, but the young practitioner will generally find more occaſion to fear the exceſſes than the deficiences of inflammation.

Old habits may in ſome inſtances be connived at, but a man ſhould conſult the records before he grants licences of this kind. Theſe are, however, digreſſions from the ſubject of imperfect union in fractures. The ſoldier was adviſed to undergo an operation for the removal of the ſplinter, but it was poſtponed. Should the enquiries which I have put forth, and the remarks of your worthy correſpondent, lead ſurgeons to avert, in any ſolitary inſtance, the misfortune of a diſunited fracture, by looking to the cauſes already aſſigned, or by fixing on more juſt and more philoſophical cauſes, the objects of my enquiry will be anſwered. The ſame motives and the ſame ſatisfaction cannot fail to prove the reward of Mr. Wilkinſon's labours.

*Soho Square,* I am, Dear Sir, &c.

*November* 5, 1801. A. CARLISLE.

